# Case report of acute myocarditis after administration of COVID-19 vaccine in Japan

**DOI:** 10.1093/ehjcr/ytab534

**Published:** 2022-01-05

**Authors:** Masato Ohnishi, Yasunori Tanaka, Sakiya Nishida, Toshiro Sugimoto

**Affiliations:** 1 Division of Cardiovascular Medicine, National Hospital Organization Higashi-Ohmi General Medical Center, Higashi-Ohmi, Japan; 2 Department of Comprehensive Internal Medicine, Shiga University of Medical Sciences, Otsu, Japan; 3 Division of General Internal Medicine, National Hospital Organization Higashi-Ohmi General Medical Center, Higashi-Ohmi, Japan

**Keywords:** myocarditis, COVID-19, vaccination

## Abstract

**Background:**

The worldwide spread of Coronavirus disease 2019 (COVID-19) is still not under control and vaccination in Japan started in February 2021, albeit later than in Europe and the United States of America. The COVID-19 vaccination frequently leads to minor adverse reactions, that may be more intense after the second dose. The number of case reports of myocarditis following COVID-19 vaccination have been recently increased.

**Case Summary:**

We report a case of a 26-year-old healthy man who presented to our hospital with chest pain on May 24 2021, 4 days after his second COVID-19 vaccination. The electrocardiogram showed ST elevation with upward concavity in I, II, aVL, aVF, V4 to V6, and small Q wave in II, III, aVF. Laboratory studies revealed elevation of troponin-I, creatine kinase, C-reactive protein and negative viral serologies. Acute aortic dissection and pulmonary thromboembolism were ruled out by contrast-enhanced thoracoabdominal computed tomography (CT). An urgent coronary angiogram was performed because an acute coronary syndrome was suspected, but no significant stenosis was found. Cardiac magnetic resonance imaging (MRI) demonstrated oedema and late gadolinium enhancement of the left ventricle in a midmyocardial and epicardial distribution.

**Discussion:**

Although the temporal association does not prove causation, the very short span between the second vaccination and the onset of myocarditis suggests that this acute myocarditis seemed to be an adverse reaction to COVID-19 vaccine. To the best of our knowledge, this is the first published case of acute myocarditis following COVID-19 vaccine in Asia.

Author Contributions: Masato Ohnishi took primary responsibility for writing this article and care of the patient, and obtained consent from the patient. Yasunori Tanaka mainly conducted cardiac catheterization and contributed to the care of the patient. Sakiya Nishida examined the patient on his first outpatient visit, made a differential diagnosis and participated in his treatment after admission. Toshiro Sugimoto provided clinical expertise and critical appraisal of the article.

**Learning points:**

・As Coronavirus disease 2019 (COVID-19) vaccination has increased, reports of adverse reactions have also increased.

・We should be aware that acute myocarditis after administration of COVID-19 vaccine may also occur even in healthy young men without immune abnormalities.

・Cardiac magnetic resonance imaging is useful in the diagnosis of acute myocarditis.

2d. Lead Author Photo

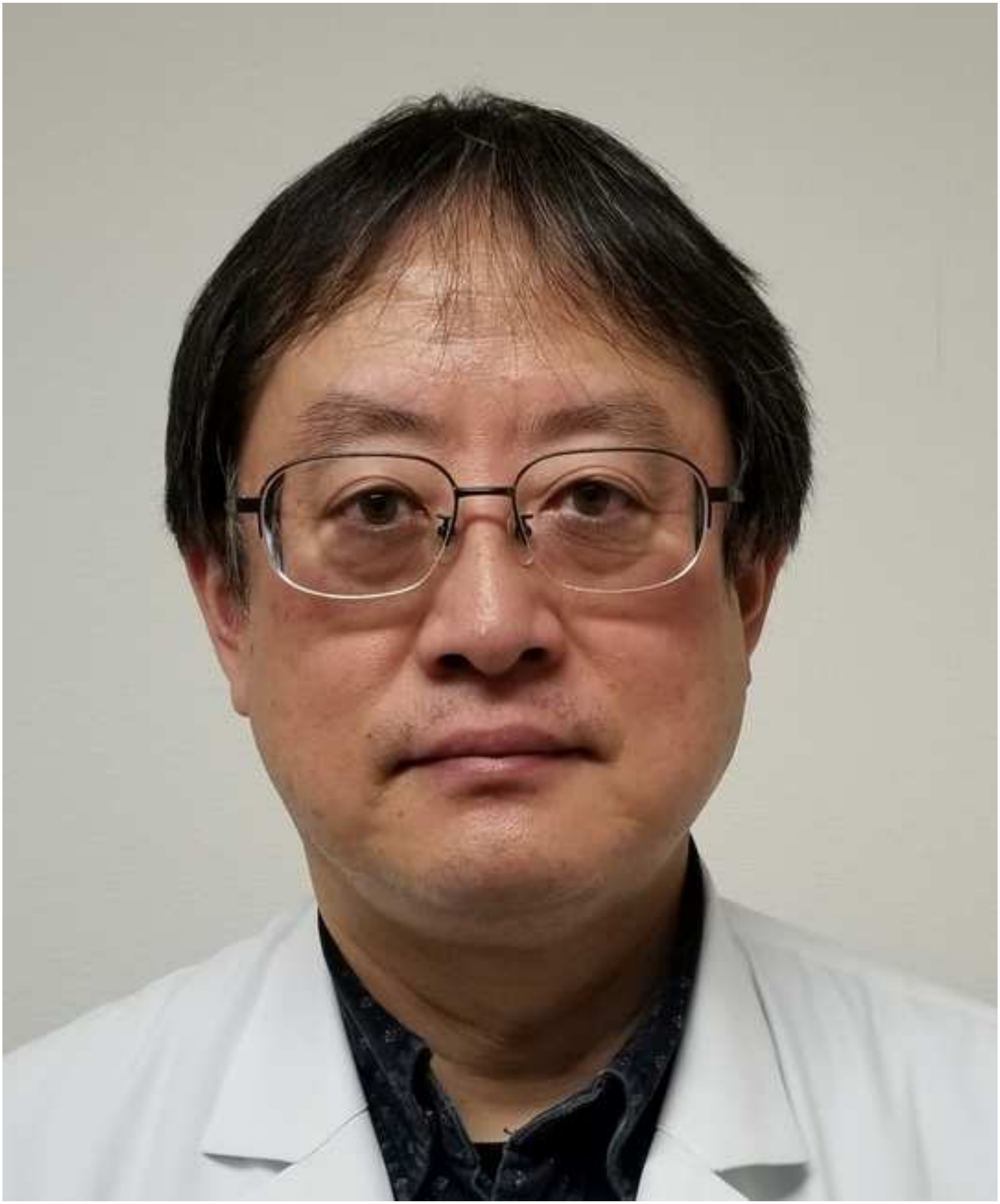

3. Learning points (1 or 2 bullet points which highlight the key learning point to the reader

Click here to access/download;3. Learning points (1 or 2 bullet points which highlight the key learning point to the

**Learning points:**

- As Coronavirus disease 2019 (COVID-19) vaccination has increased, reports of adverse reactions have also increased.
- We should be aware that acute myocarditis after administration of COVID-19 vaccine may also occur even in healthy young men without immune abnormalities.
- Cardiac magnetic resonance imaging is useful in the diagnosis of acute myocarditis.

**Acknowledgement**

We thank Dr Hiroshi Sakai (Department of Cardiology, Shiga University of Medical Sciences, Otsu, Japan) for arranging the cardiac MRI and Dr Yukihiro Nagatani (Department of Radiology, Shiga University of Medical Sciences, Otsu, Japan) for reading the cardiac MRI.

Click here to access/download;5. Organised Timeline for case report (table or figure);Timeline(revised).docx

**Timeline**

| Time | Event |
|---|---|
| 30 April | First dose of COVID-19 vaccine. |
| 21 May | Second dose of COVID-19 vaccine. |
| 22 May | Fever, headache, appetite loss, general malaise, and shoulder stiffness, which was treated with acetaminophen. |
| 23 May | Onset of chest pain and persistent fever, headache, appetite loss. |
| 24 May | The patient visited outpatient clinic and was admitted to the high care unit (HCU); elevated markers of myocardial damage such as high-sensitivity troponin I, Creatine Kinase, C-reactive protein, diffuse ST-segment elevation on electrocardiogram (ECG). An urgent coronary angiogram showed no significant stenosis. anti-inflammatory drugs were started and the patient's symptoms improved. |
| 25-26 May | The patient was haemodynamically stable and asymptomatic. A subsequent ECG showed partial resolution of the ST changes and a trend towards improvement. |
| 27 May | The patient was discharged in order to perform cardiac magnetic resonance imaging (MRI). |
| 31 May | The patient visited outpatient clinic with mild general fatigue. Cardiac MRI showed myocardial late gadolinium enhancement with epicardial predominance in the antero-septal, inferior and lateral walls of the basal segment and apex, which were consistent with acute myocarditis. |
| 3 June | The patient recovered and returned to work. |
| 14 June | Despite no deterioration in cardiac function, the patient complained of appetite loss and fatigue on exertion, was diagnosed as post-vaccination syndrome and was absent from work for a month. |
| 12 July | The patient recovered to some extent and returned to work. |
| 29 July | The patient complained of general malaise and sleep disturbance, was diagnosed as depressive state and is currently on leave. |

## Introduction

The worldwide spread of Coronavirus disease 2019 (COVID-19) is still not under control and vaccination in Japan started in February 2021, albeit later than in Europe and the United States of America. Adverse reactions to vaccination are usually not very significant. However, with the second dose of the COVID-19 vaccine, mild adverse reactions are often more severe and more serious adverse reactions have been reported [1]. Very recently, several cases have been published of myocarditis associated with severe acute respiratory syndrome coronavirus 2 (SARS-CoV-2) vaccines such as BNT162b2 (Pfizer-BioNTech) and mRNA-1273 (Moderna) [2, 3]. At time of writing, there are no published case reports of COVID-19 vaccine related myocarditis in Asia.

## Case presentation

We present the case of a 26-year-old male clinical engineer working in our hospital who came to our hospital complaining of acute substernal chest pain on May 21 2021, 4 days after his second dose of BNT162b2 vaccine. The patient was an Asians of Mongoloid descent and had no previous medical history. The patient gave informed consent for the write-up and publication of this clinical case. The electrocardiogram (ECG) on arrival showed small Q waves in II, III, aVF, ST elevation with upward concavity in I, II, aVL, aVF and V4-V6, and ST depression and deep negative T wave in aVR. (Figure 1A). Laboratory studies revealed elevated markers of myocardial damage such as high-sensitivity troponin I (hs-TnI, 5362.4pg/mL, referential range (RR) 0-26.2pg/mL), Creatine Kinase (CK, 332U/L, RR 59-248U/L), C-reactive protein (7.57mg/dL, RR 0-0.14mg/dL), and a negative polymerase chain reaction (PCR) test for COVID-19. The plasma electrolytes such as sodium and potassium were within normal limits. Viral studies including coxsackie group B viruses and echoviruses were all negative. Autoimmune studies such as antinuclear antibodies and thyroid hormones were within normal limits. There were no significant findings on chest X-ray. Transthoracic echocardiography on admission revealed good left ventricular function, no regional wall motion abnormalities, no significant valvular disease and no pericardial effusion (Figure 2-A), his left ventricular ejection fraction (EF) was 53%, within normal limits, and further improved to 65% during his hospitalization. Acute aortic dissection and pulmonary thromboembolism were ruled out by contrast-enhanced thoracoabdominal computed tomography (CT). Thoracic CT showed no pericardial effusion or ventricular wall thickening (Figure 2-B). An urgent coronary angiogram was performed because an acute coronary syndrome was suspected, but no significant stenosis of the coronary arteries was found except for an incidental finding of coronary artery (left anterior descending artery) to pulmonary artery fistula (Figure 2-C, D). The patient was admitted to the high care unit, although he remained hemodynamically stable and asymptomatic. Subsequent ECGs showed partial resolution of the ST changes and a trend towards improvement (Figure 1B). No endocardial biopsy was performed because of the patient's low risk and good course. The patient was discharged after 4 days with resolution of symptoms and improvement in inflammatory response and myocardial injury markers (Table). Cardiac magnetic resonance imaging (MRI) examination, which was obtained on discharge 6 days after second dose of vaccination, showed maintained systolic function, increased myocardial and pericardial signal intensity on short T1 inversion recovery (STIR) sequences and slight elevated signals of the myocardium on dynamic contrast-enhanced sequences, suggesting increased vascular flow. Fast imaging employing steady state acquisition (FIESTA) sequences, which were performed 15 minutes after intravenous administration of gadolinium, demonstrated sub-epicardial enhancement of the myocardium, which was considered consistent with acute myocarditis. T1-weighted (T1WI) sequences also showed late subepicardial enhancement in the anterior wall and interventricular septum near the apex (Figure 3). These findings were considered consistent with acute myocarditis. The patient was initially treated with oral acetaminophen, antitussives and no steroids, with progressive resolution of his symptoms. The patient has scheduled outpatient cardiology follow-up. On the fourth day after discharge, the patient presented to the outpatient clinic with only mild general malaise and returned to work three days later. However, despite no deterioration in cardiac function, the patient complained of appetite loss and fatigue on exertion, was diagnosed as post-vaccination syndrome and was absent from work for a month. The patient recovered to some extent and returned to work, but two weeks later he complained of general malaise and sleep disturbance, was diagnosed with situational depression and is currently on leave to see a psychotherapist.

**Discussion**

In the present case, a healthy young adult male developed acute myocarditis after COVID-19 vaccination. The diagnosis of myocarditis was confirmed by cardiac MRI with late gadolinium enhancement sequence, although no endocardial biopsy was performed. Non-invasive imaging modalities such as MRI, CT, and echocardiogram are useful for diagnosis and are of benefit to patients. In addition, FIESTA with superior temporal resolution is thought to provide better depiction of late enhancement effect in the myocardium than conventional T1WI. The temporal association between vaccination and the onset of symptoms, and the exclusion of other acute cardiac diseases, suggested that this acute myocarditis seemed to be an adverse reaction to the BNT162b2 vaccine.

Although the temporal association does not prove causation, the very short span between the second vaccination and the onset of myocarditis suggests a possible relationship. Since April 2021, the number of cases of cardiac inflammation (myocarditis and pericarditis) after mRNA COVID-19 vaccination (Pfizer-BioNTech and Moderna) have been increasing in the United States [4]. The Centers for Disease Control and Prevention (CDC) has announced that more than 1,000 cases of myocarditis/pericarditis after mRNA vaccination have been reported to the Vaccine Adverse Event Reporting System (VAERS) on June 25, 2021. Myocarditis/pericarditis develops with sudden chest pain 1-5 days after vaccination, is more often in males under 30 years of age, and is more often after the second vaccination, so this case shows a typical pattern of onset. Although the onset of the disease may be mediated by the immune system, it is currently unclear what immune response to the mRNA vaccine leads to the development of myocarditis/pericarditis. A possible pathogenic mechanism is direct spike mediated toxicity. Myocarditis/pericarditis has also been reported as a complication of SARS-CoV-2 infection and may involve an immune response to spike proteins, but unfortunately we did not perform quantitative SARS-CoV-2 antibody assays in this case. Another hypothesis is a delayed hypersensitivity reaction and eosinophilic myocarditis that develops directly after vaccination [5]. As shown in the Table, the changes in eosinophils in peripheral blood showed a low level of 0.4% (30 cells/μL) on Day1, the day of admission, followed by a gradual increase and a mild increase to 4.6% (200 cells/μL) on Day4. We speculate that an eosinophilic infiltration of the myocardial tissue may have occurred immediately after the second vaccination, but this cannot be proven as we have not performed a myocardial biopsy.

In Japan, priority vaccination of healthcare providers first started in February 2021, and vaccination at our hospital started in May. In June, vaccination of elderly people aged 75 years and over started on a priority basis, but vaccination of the general young population, especially those in their twenties, is still largely unavailable. Therefore, as vaccination spreads to the younger generation, there is a concern that cases of myocarditis and pericarditis may increase. The Ministry of Health, Labour and Welfare announced 12 cases of Myocarditis/pericarditis as of 2 July, and the Japanese Circulation Society issued a statement on "Acute myocarditis and acute pericarditis after vaccination with novel coronaviruses" on 21 July. We healthcare providers have to pay attention to this issue as vaccination in Japan will progress from the elderly to the younger generation. However, it appears that there is a significantly higher risk of cardiac involvement from COVID-19 infection compared to COVID-19 vaccination [6]. We therefore believe that COVID-19 vaccination plays an important role in herd immunity and overall provide more benefit than harm.

The patient was a medical professional and was well aware that myocarditis can be fatal in severe cases, which may have been traumatic and led to depressive state. It is a priority to provide psychological support for the post-traumatic stress disorder (PTSD)-like depression which can occur as an adverse complication of the vaccine.

In conclusion, to the best of our knowledge, this is the first published case of acute myocarditis as an adverse reaction to SARS-CoV-2 vaccine in Asia.

**Conflict of interest:** None declared.

**Funding:** None declared.

Figure 1.

(A) electrocardiogram (ECG) showing small Q waves in II, III, aVF, ST elevation with upward concavity in I, II, aVL, aVF and V4-V6, and ST depression and deep negative T wave in aVR.

(B) serial changes in ECG showing negative and or flattened T waves in I, II, aVL, aVF, V4-V6.

Figure 2.

Echocardiogram and thoracic CT showed no pericardial effusion or ventricular wall thickening (A, B). Coronary angiography demonstrated no significant stenosis of the coronary arteries except for a coronary artery (left anterior descending) to pulmonary artery fistula (arrow) (C, D).

Figure 3. Cardiac magnetic resonance images.

Short T1 inversion recovery axis image acquired along the basal short-axis view demonstrates increased subepicardial and mesocardial signal intensity of the antero-septal myocardial segments (A). Dynamic contrast-enhanced sequence demonstrates slight elevated signals of the antero-septal myocardial segments (B). Fast imaging employing steady state acquisition (FIESTA) sequences acquired along the basal short-axis view (C) and four-chamber view (D) demonstrate myocardial late gadolinium enhancement with epicardial predominance in the antero-septal, inferior and lateral walls of the basal segment and apex.

Click here to access/download;13. Table;Table(EHJ)revised.docx

Table

| | Day1 | Day2 | Day3 | Day4 | Day8 |
|---|---|---|---|---|---|
| CRP (mg/dL) [0-0.14] | 7.57 | 4.24 | 1.57 | 0.70 | 0.13 |
| HS-Tn-I (pg/mL) [0-26.2] | 5362.4 | 4426.0 | 2768.1 | | |
| WBC (/μL) [3300-8600] | 8560 | 5440 | 4460 | 4320 | 5600 |
| Eosinophil (/μL) [2-4%] | 30 (0.4%) | 140 (2.6%) | 170 (3.8%) | 200 (4.6%) | 190 (3.4%) |
| *CK (U/L) [59-248]* | *332* | *315* | *96* | *55* | *39* |
| AST (U/L) [13-30] | 44 | 48 | 29 | 27 | 25 |
| ALT (U/L) [10-42] | 38 | 33 | 30 | 37 | 47 |
| LD (U/L) [124-222] | 226 | 219 | 226 | 200 | 191 |
| BNP (pg/mL) [0-18.4] | 20.2 | | | | |

Markers of myocardial injury and inflammatory response have improved within one week.

CRP, C-reactive protein; HS-Tn-I, high-sensitivity troponin-I; WBC, white blood cell; CK, creatine kinase; AST, aspartate aminotransferase; ALT, alanine aminotransferase; LD, lactate dehydrogenase; BNP, brain natriuretic peptide.

Figure (colour)

Click here to access/download;Figure (colour);Figure(EHJ)revised.docx

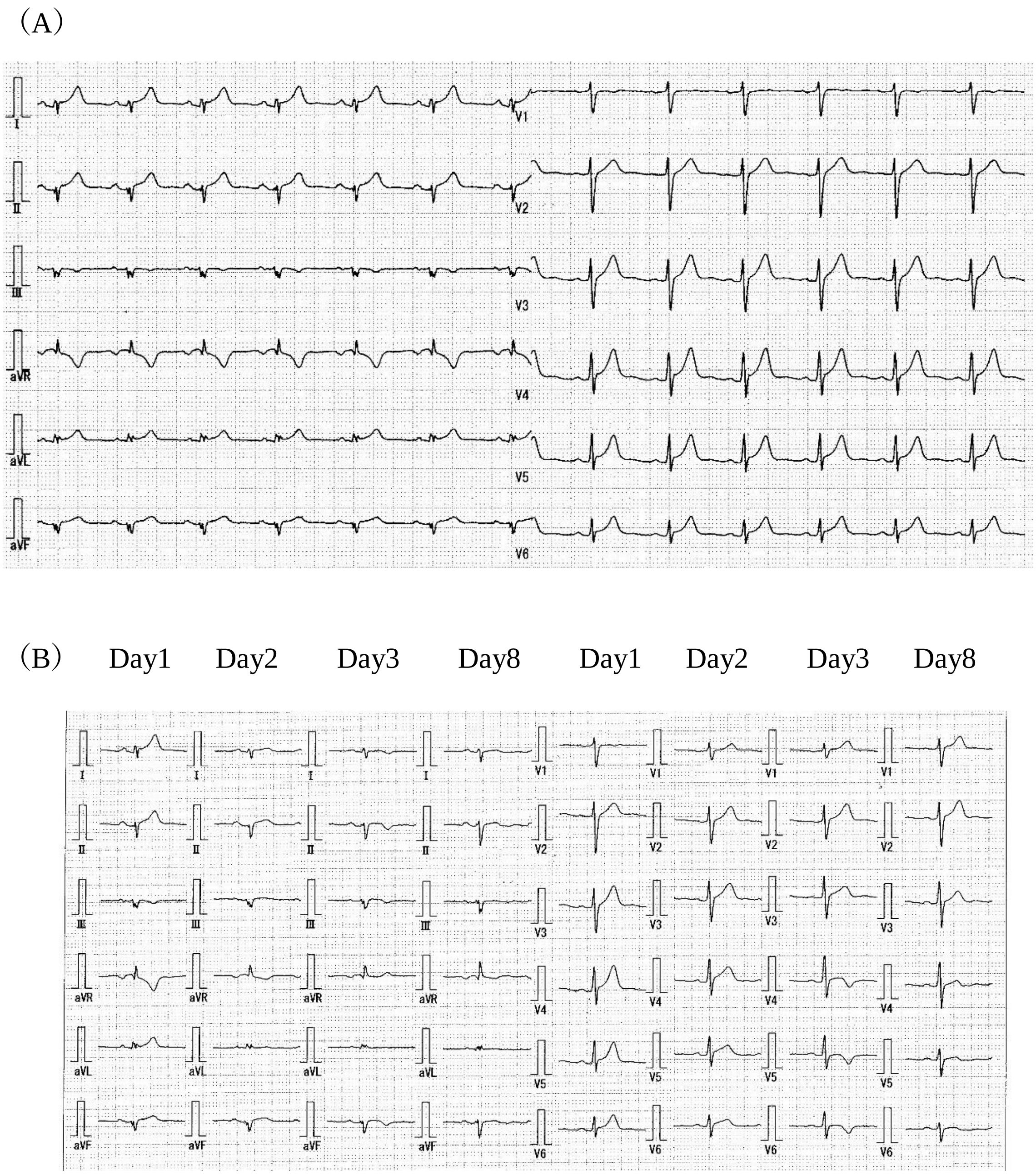

Figure 1.

(A) electrocardiogram (ECG) showing small Q waves in II, III, aVF, ST elevation with upward concavity in I, II, aVL, aVF and V4-V6, and ST depression and deep negative T wave in aVR. (B) serial changes in ECG showing negative and or flattened T waves in I, II, aVL, aVF, V4-V6.

Figure 2.

Echocardiogram and thoracic CT showed no pericardial effusion or ventricular wall thickening (A, B). Coronary angiography demonstrated no significant stenosis of the coronary arteries except for a coronary artery (left anterior descending) to pulmonary artery fistula (arrow) (C, D).

Figure 3. Cardiac magnetic resonance images.

Short T1 inversion recovery axis image acquired along the basal short-axis view demonstrates increased subepicardial and mesocardial signal intensity of the antero-septal myocardial segments (A). Dynamic contrast-enhanced sequence demonstrates slight elevated signals of the antero-septal myocardial segments (B). Fast imaging employing steady state acquisition (FIESTA) sequences acquired along the basal short-axis view (C) and four-chamber view (D) demonstrate myocardial late gadolinium enhancement with epicardial predominance in the antero-septal, inferior and lateral walls of the basal segment and apex.

## Supplementary Material

ytab534_Supplementary_Data

