# Supplementary material for: Case report of acute myocarditis after administration of COVID-19 vaccine in Japan
Source: Eur Heart J Case Rep. 2022 Jan 5:ytab534. doi: 10.1093/ehjcr/ytab534 (PMC8755377; doi:10.1093/ehjcr/ytab534)
Supplement: ytab534_Supplementary_Data [file ytab534_supplementary_data.zip › Suppl/EHJ-CR-Slide-Set revised.pptx]

## Slide 1
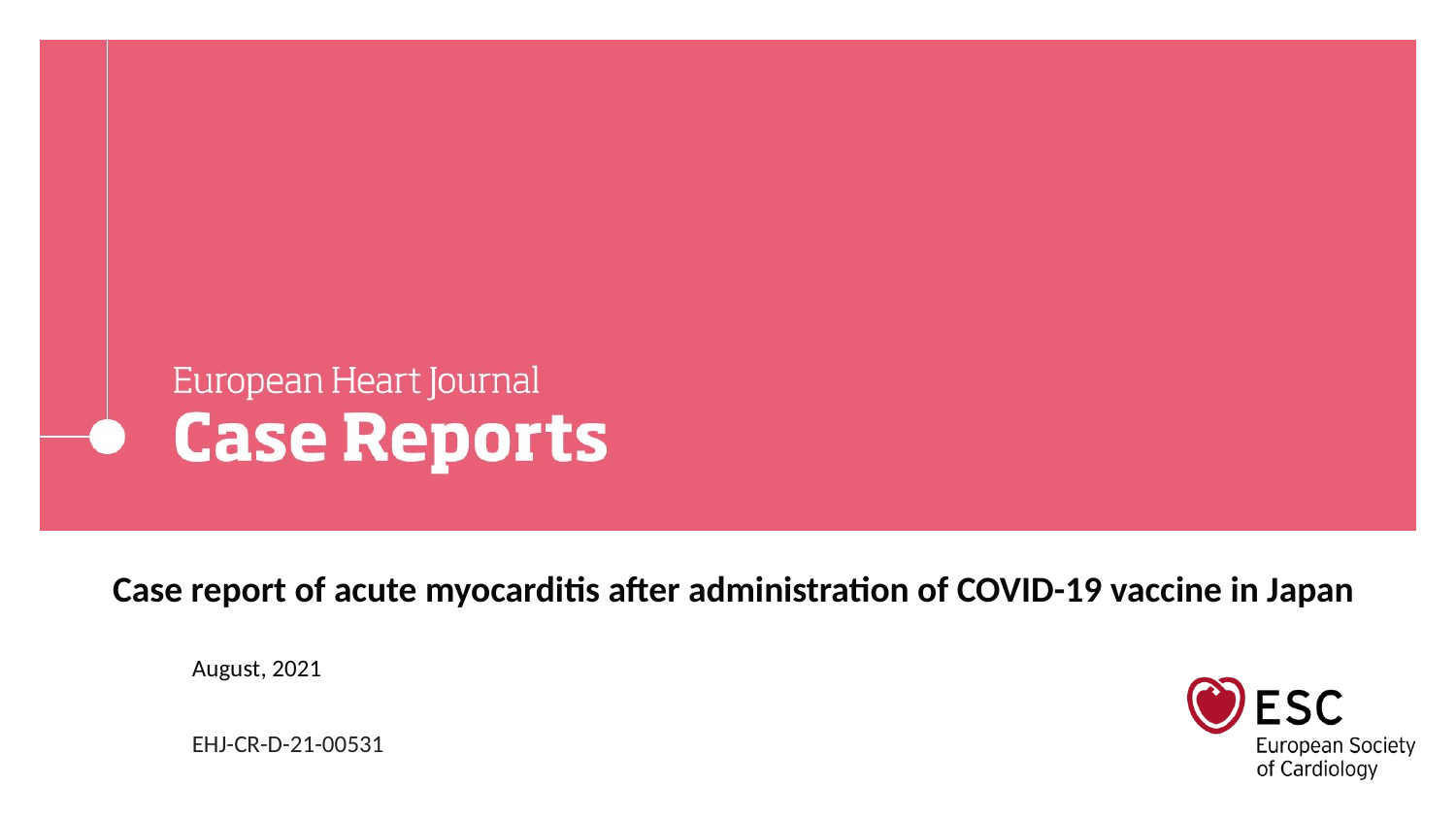

# Case report of acute myocarditis after administration of COVID-19 vaccine in Japan
August, 2021
EHJ-CR-D-21-00531

## Slide 2
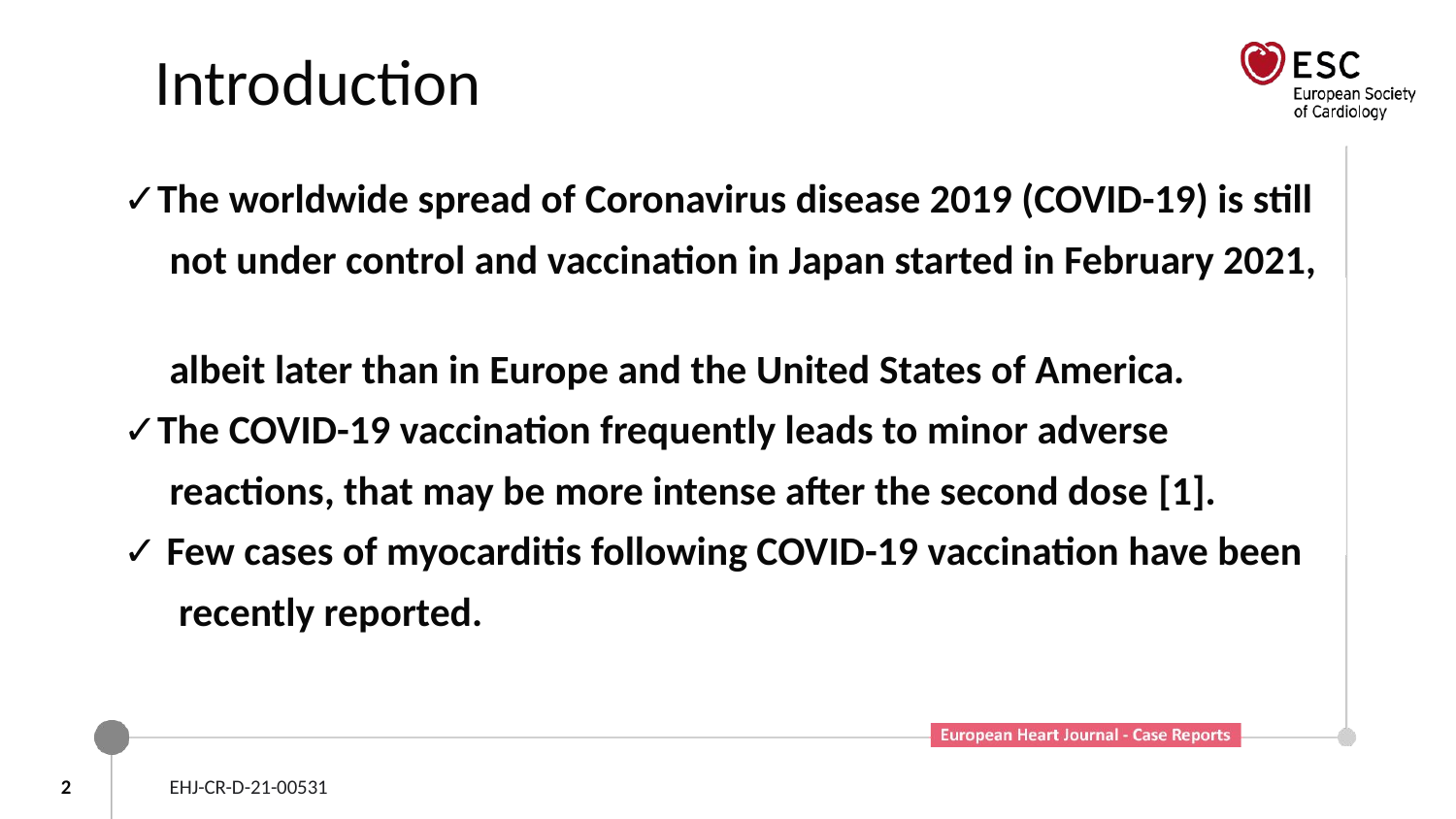

# Introduction
✓The worldwide spread of Coronavirus disease 2019 (COVID-19) is still
 not under control and vaccination in Japan started in February 2021,
 albeit later than in Europe and the United States of America.
✓The COVID-19 vaccination frequently leads to minor adverse
 reactions, that may be more intense after the second dose [1].
✓ Few cases of myocarditis following COVID-19 vaccination have been
 recently reported.
2
EHJ-CR-D-21-00531

## Slide 3
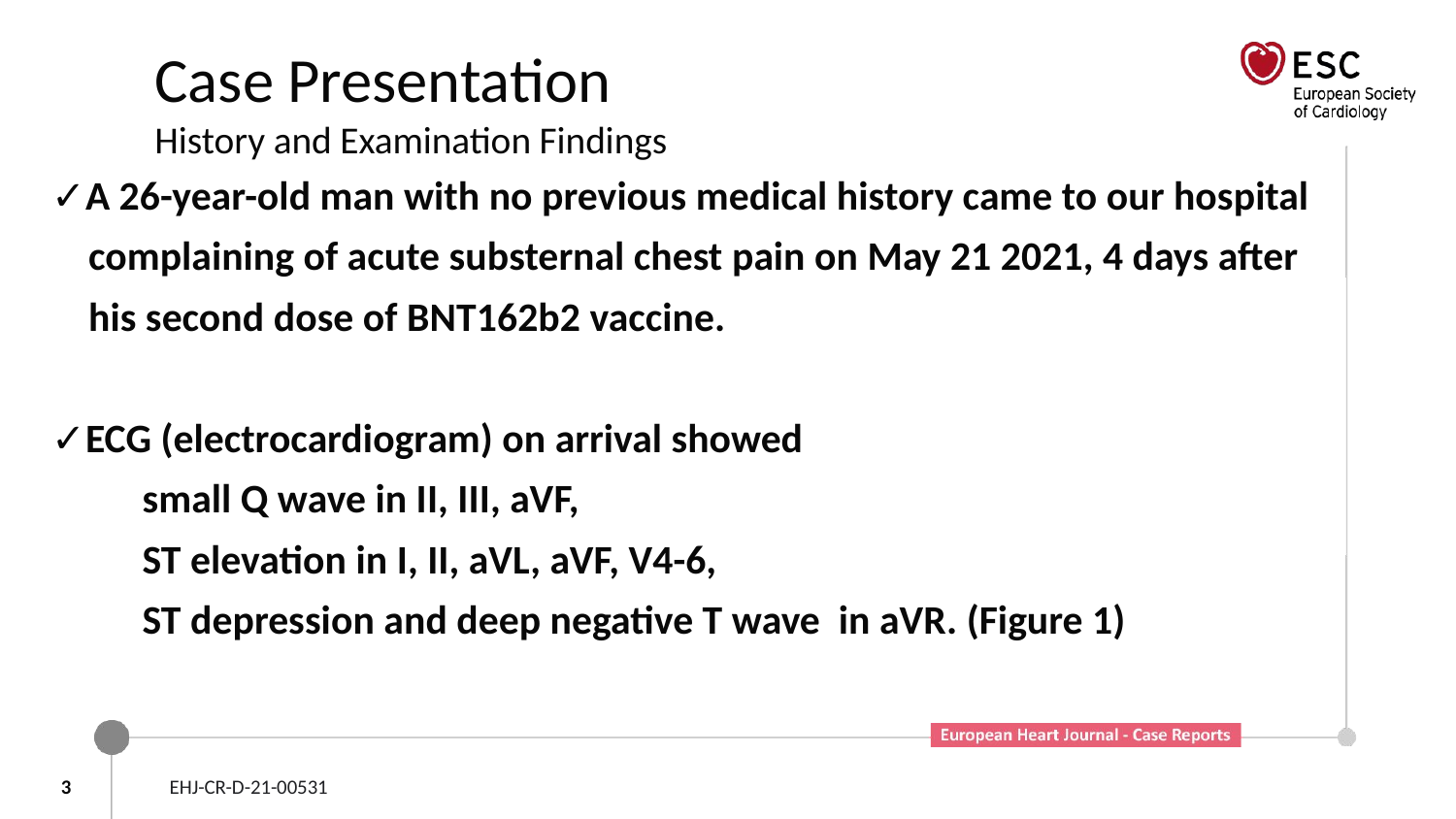

# Case Presentation History and Examination Findings
✓A 26-year-old man with no previous medical history came to our hospital
 complaining of acute substernal chest pain on May 21 2021, 4 days after
 his second dose of BNT162b2 vaccine.
✓ECG (electrocardiogram) on arrival showed
　　small Q wave in II, III, aVF,
　　ST elevation in I, II, aVL, aVF, V4-6,
　　ST depression and deep negative T wave in aVR. (Figure 1)
3
EHJ-CR-D-21-00531

## Slide 4
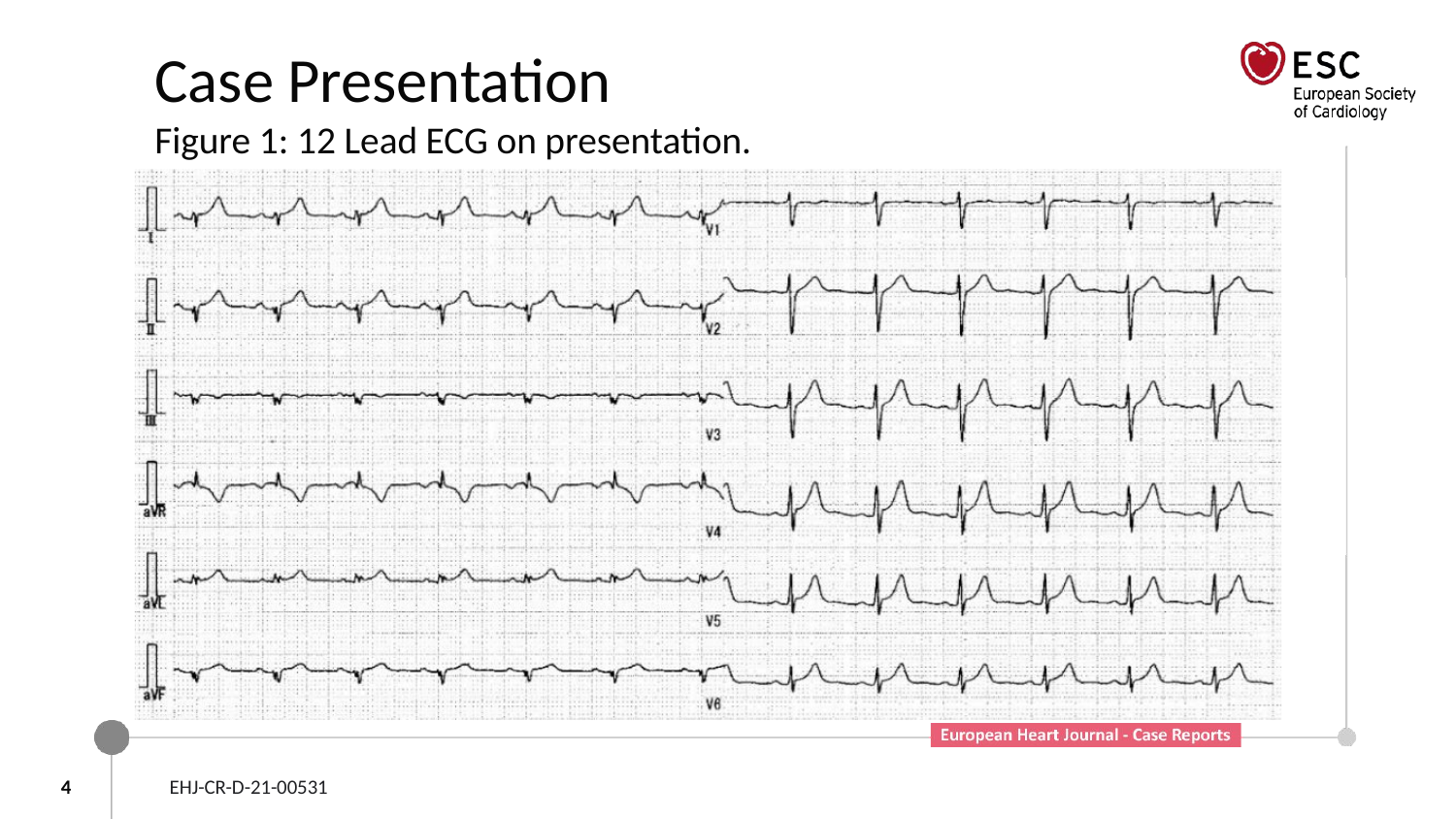

# Case Presentation Figure 1: 12 Lead ECG on presentation.
4
EHJ-CR-D-21-00531

## Slide 5
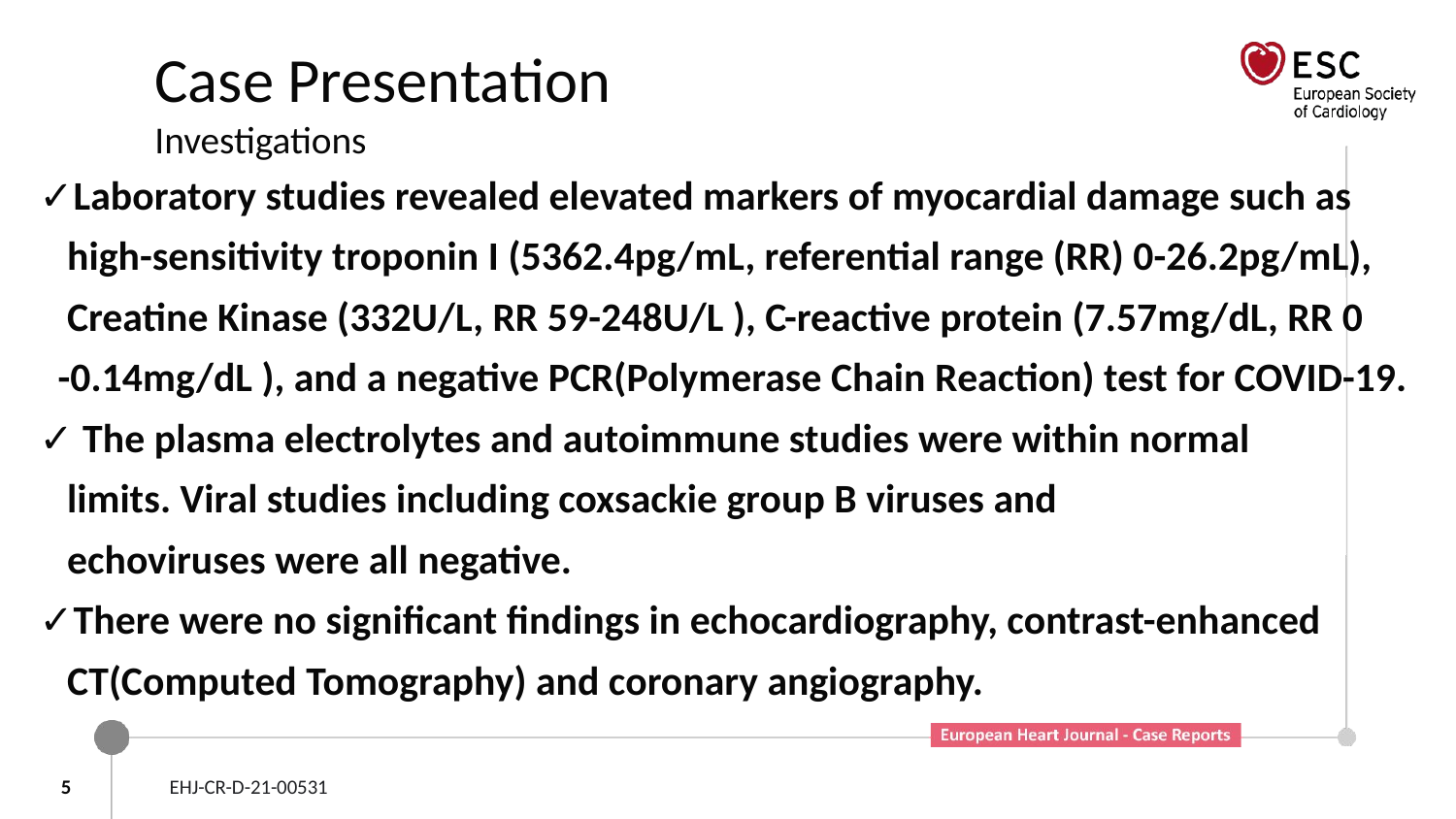

# Case Presentation Investigations
✓Laboratory studies revealed elevated markers of myocardial damage such as
 high-sensitivity troponin I (5362.4pg/mL, referential range (RR) 0-26.2pg/mL),
 Creatine Kinase (332U/L, RR 59-248U/L ), C-reactive protein (7.57mg/dL, RR 0
 -0.14mg/dL ), and a negative PCR(Polymerase Chain Reaction) test for COVID-19.
✓ The plasma electrolytes and autoimmune studies were within normal
 limits. Viral studies including coxsackie group B viruses and
 echoviruses were all negative.
✓There were no significant findings in echocardiography, contrast-enhanced
 CT(Computed Tomography) and coronary angiography.
5
EHJ-CR-D-21-00531

## Slide 6
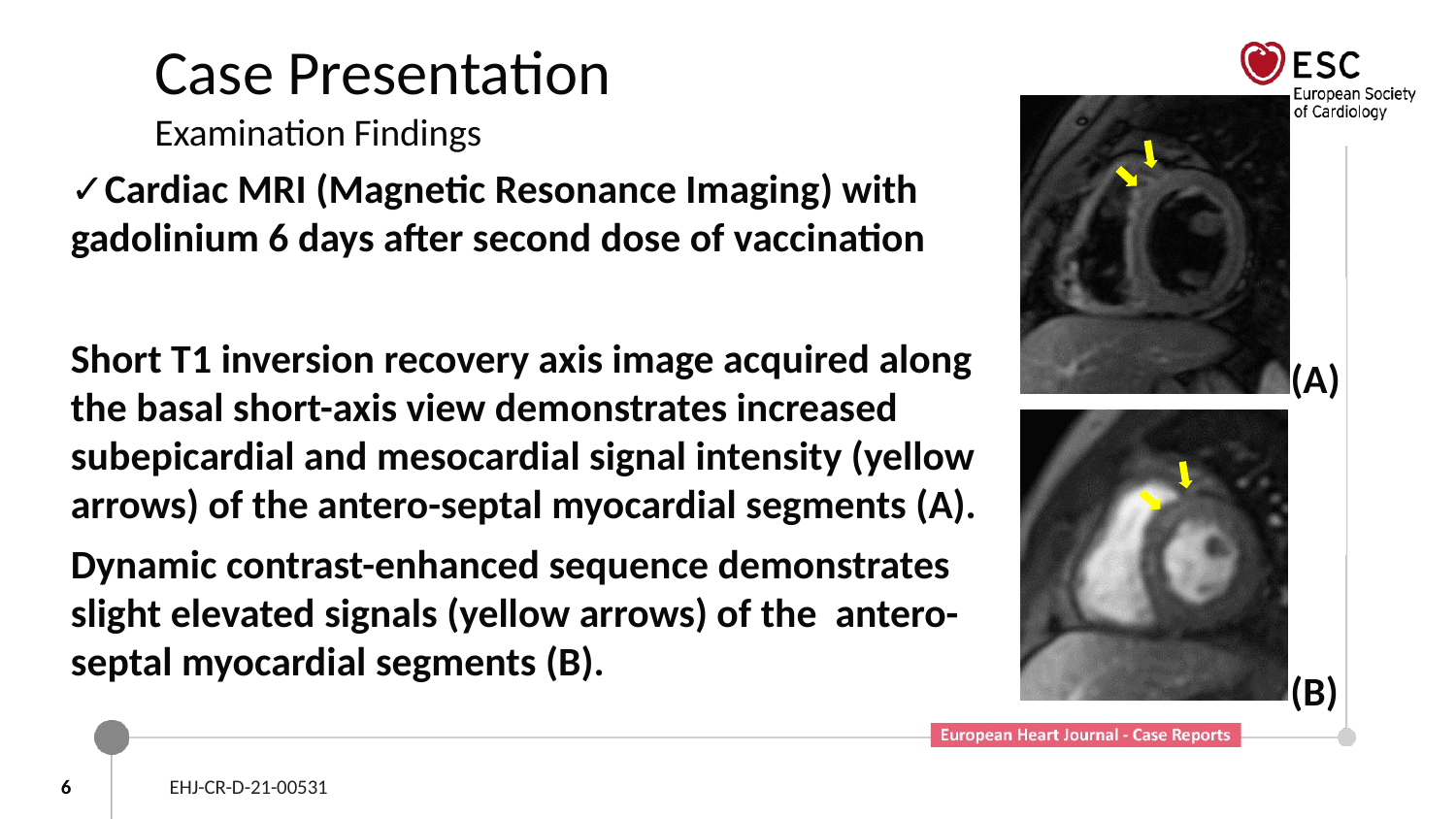

# Case Presentation Examination Findings
✓Cardiac MRI (Magnetic Resonance Imaging) with gadolinium 6 days after second dose of vaccination
Short T1 inversion recovery axis image acquired along the basal short-axis view demonstrates increased　subepicardial and mesocardial signal intensity (yellow arrows) of the antero-septal myocardial segments (A).
Dynamic contrast-enhanced sequence demonstrates　slight elevated signals (yellow arrows) of the antero-septal myocardial segments (B).
(A)
(B)
6
EHJ-CR-D-21-00531

## Slide 7
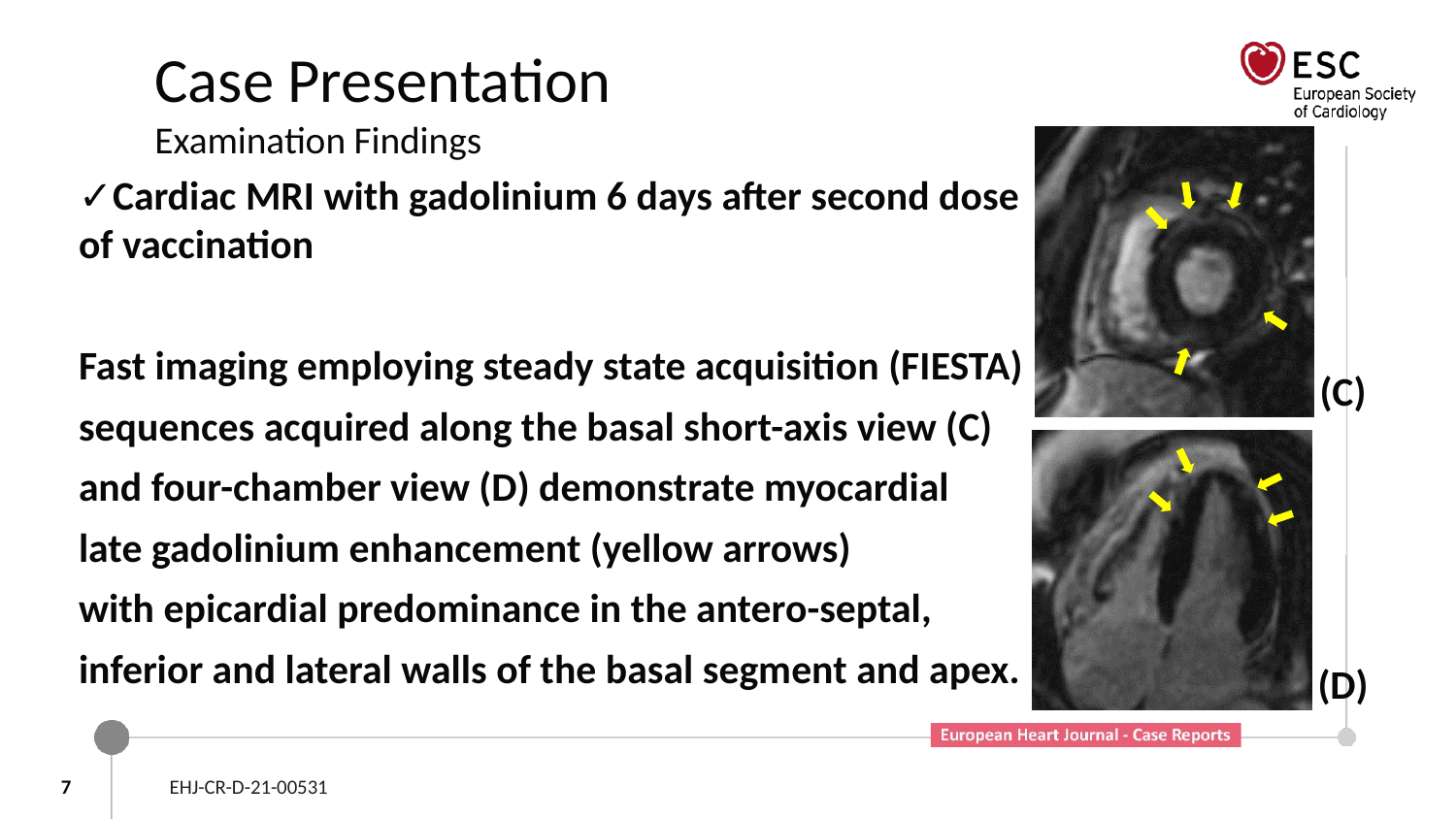

# Case Presentation Examination Findings
✓Cardiac MRI with gadolinium 6 days after second dose of vaccination
Fast imaging employing steady state acquisition (FIESTA)
sequences acquired along the basal short-axis view (C)
and four-chamber view (D) demonstrate myocardial
late gadolinium enhancement (yellow arrows)
with epicardial predominance in the antero-septal,
inferior and lateral walls of the basal segment and apex.
(C)
(D)
7
EHJ-CR-D-21-00531

## Slide 8
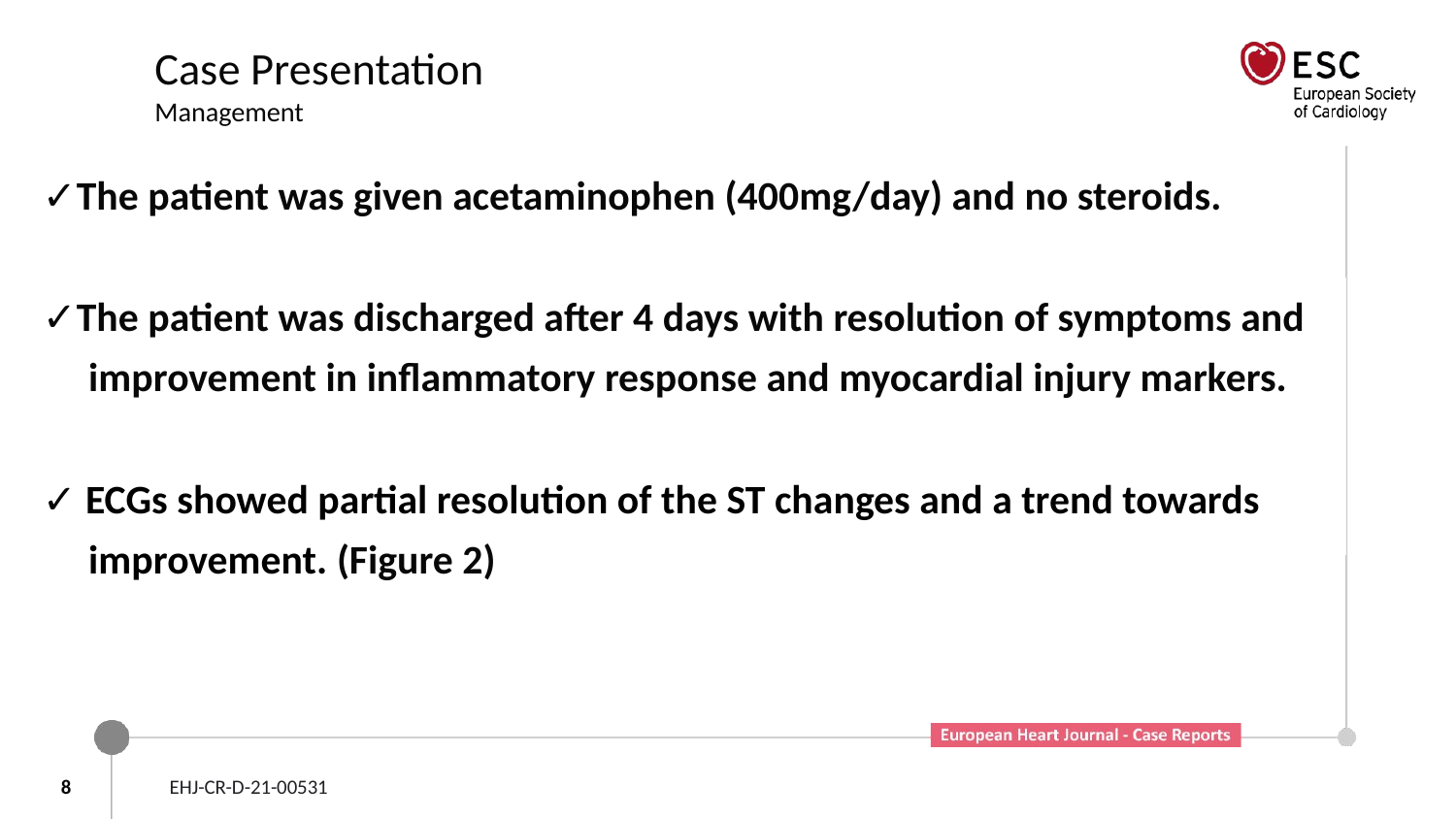

# Case Presentation Management
✓The patient was given acetaminophen (400mg/day) and no steroids.
✓The patient was discharged after 4 days with resolution of symptoms and
 improvement in inflammatory response and myocardial injury markers.
✓ ECGs showed partial resolution of the ST changes and a trend towards
 improvement. (Figure 2)
8
EHJ-CR-D-21-00531

## Slide 9
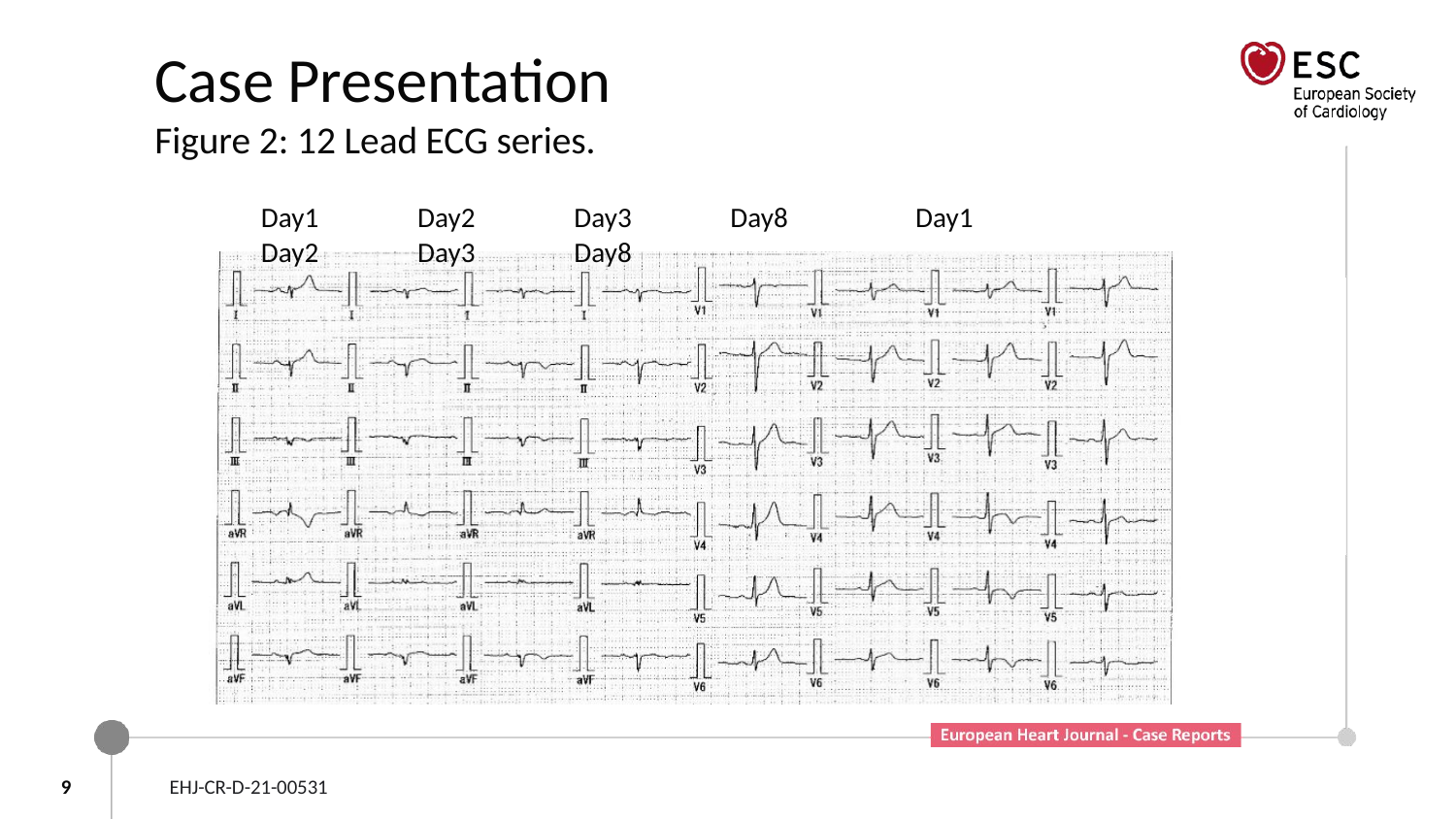

# Case Presentation Figure 2: 12 Lead ECG series.
Day1　　　Day2　　　Day3　　　Day8　　　　Day1　　　Day2　　　Day3　　　Day8
9
EHJ-CR-D-21-00531

## Slide 10
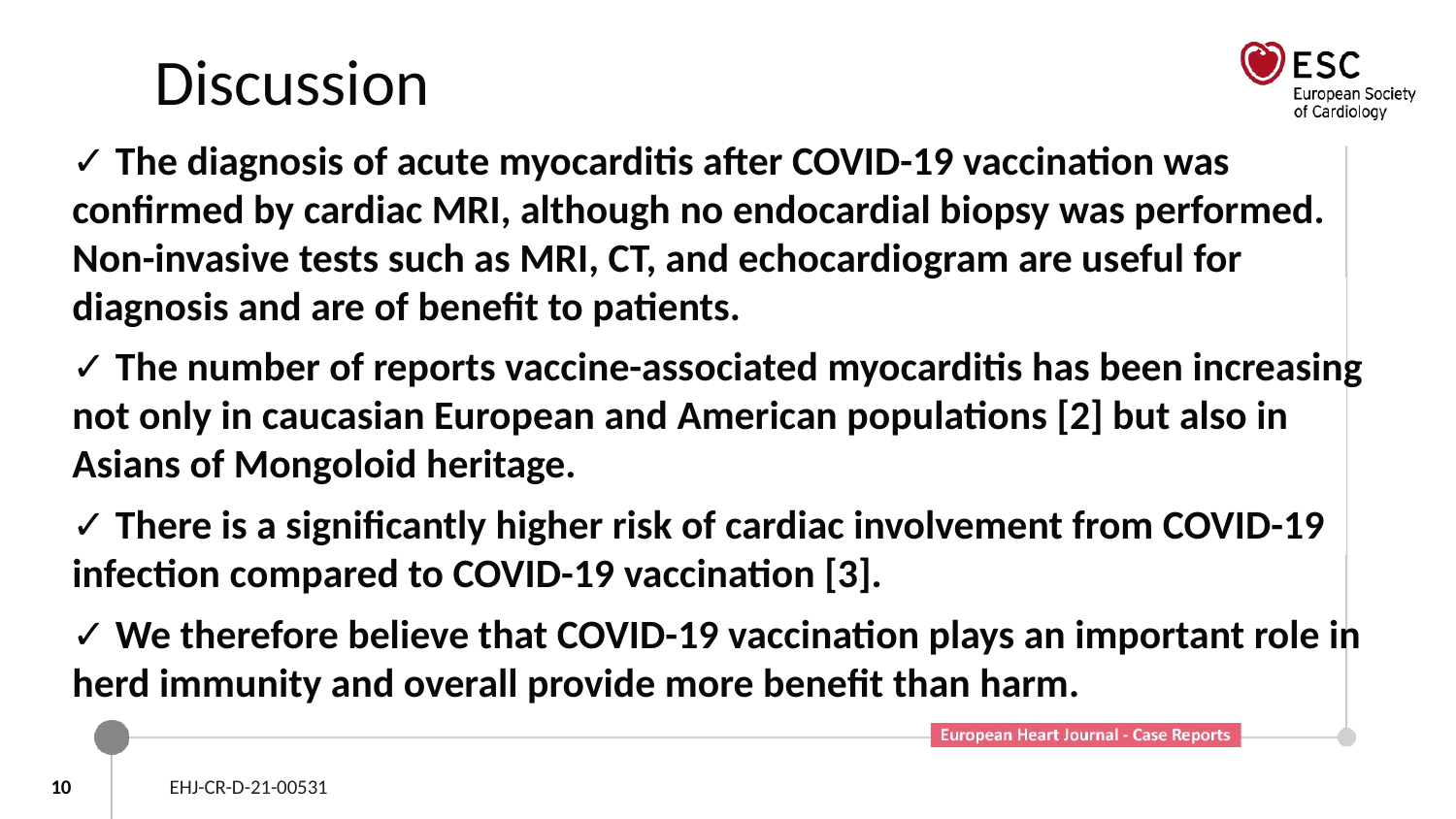

# Discussion
✓ The diagnosis of acute myocarditis after COVID-19 vaccination was confirmed by cardiac MRI, although no endocardial biopsy was performed. Non-invasive tests such as MRI, CT, and echocardiogram are useful for diagnosis and are of benefit to patients.
✓ The number of reports vaccine-associated myocarditis has been increasing not only in caucasian European and American populations [2] but also in Asians of Mongoloid heritage.
✓ There is a significantly higher risk of cardiac involvement from COVID-19 infection compared to COVID-19 vaccination [3].
✓ We therefore believe that COVID-19 vaccination plays an important role in herd immunity and overall provide more benefit than harm.
10
EHJ-CR-D-21-00531

## Slide 11
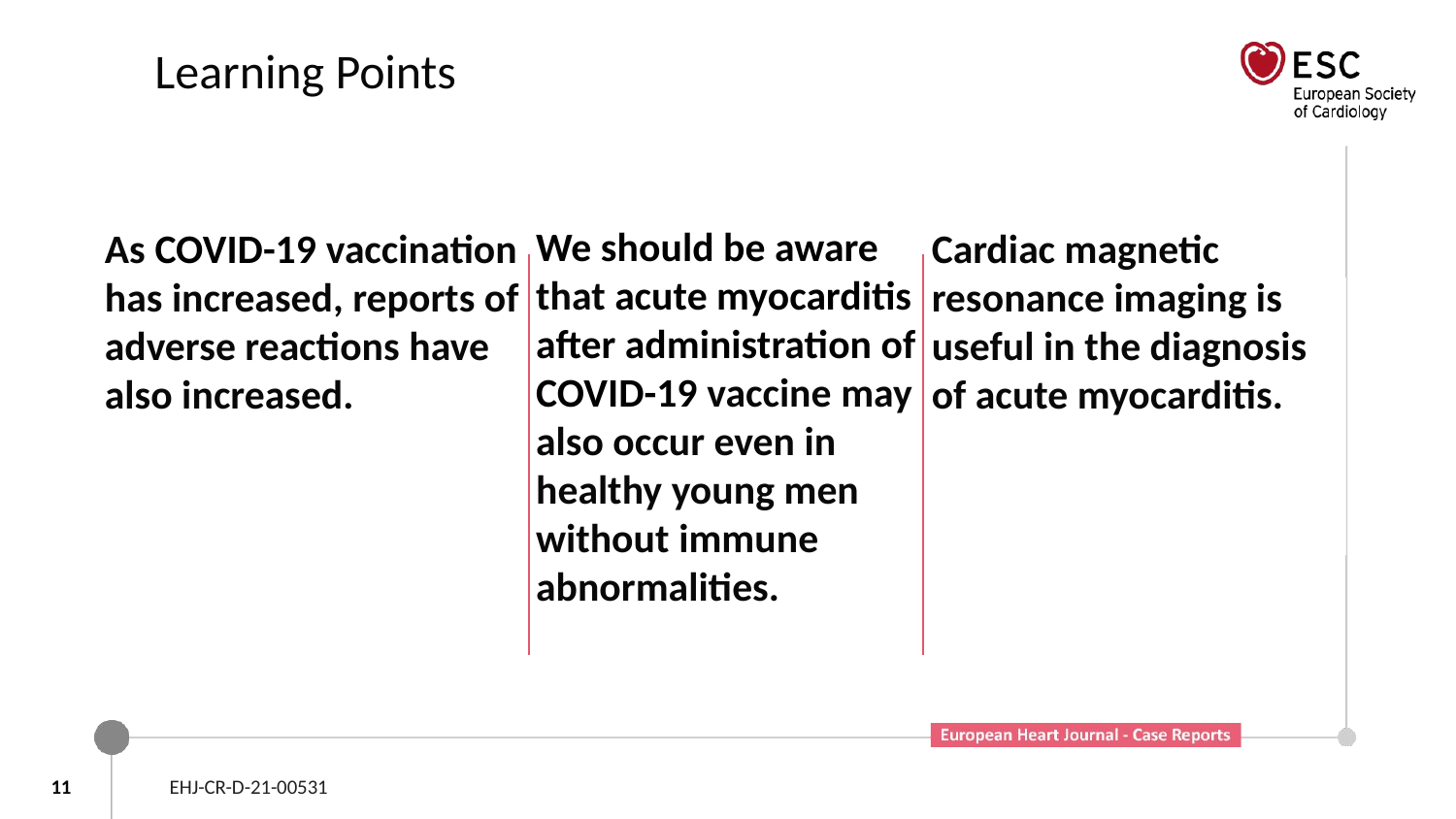

# Learning Points
We should be aware that acute myocarditis after administration of COVID-19 vaccine may also occur even in healthy young men without immune abnormalities.
As COVID-19 vaccination has increased, reports of adverse reactions have also increased.
Cardiac magnetic resonance imaging is useful in the diagnosis of acute myocarditis.
11
EHJ-CR-D-21-00531

## Slide 12
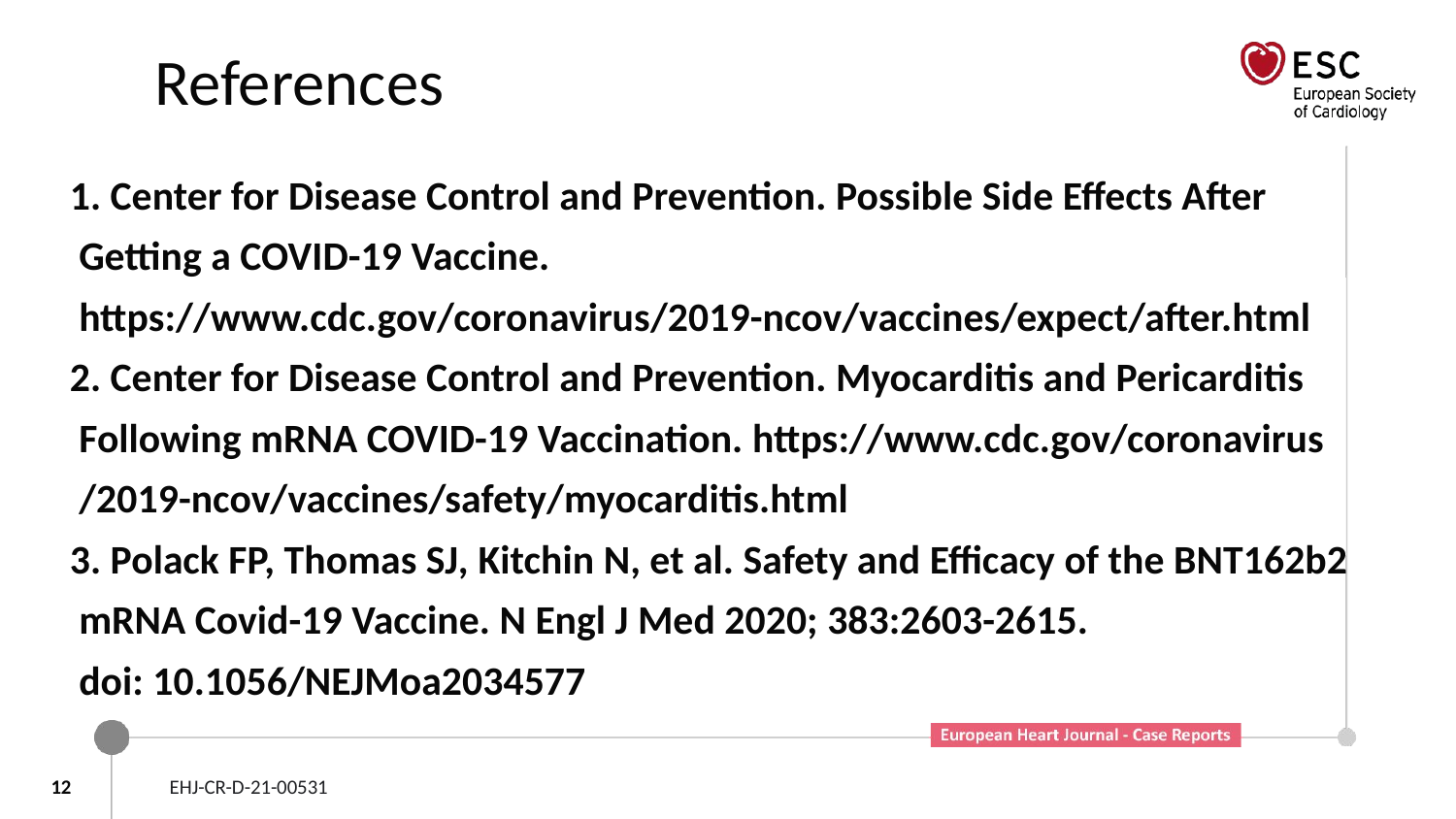

# References
1. Center for Disease Control and Prevention. Possible Side Effects After
 Getting a COVID-19 Vaccine.
 https://www.cdc.gov/coronavirus/2019-ncov/vaccines/expect/after.html
2. Center for Disease Control and Prevention. Myocarditis and Pericarditis
 Following mRNA COVID-19 Vaccination. https://www.cdc.gov/coronavirus
 /2019-ncov/vaccines/safety/myocarditis.html
3. Polack FP, Thomas SJ, Kitchin N, et al. Safety and Efficacy of the BNT162b2
 mRNA Covid-19 Vaccine. N Engl J Med 2020; 383:2603-2615.
 doi: 10.1056/NEJMoa2034577
12
EHJ-CR-D-21-00531
